# Phenotypic Changes in Diabetic Neuropathy Induced by a High-Fat Diet in Diabetic C57Bl/6 Mice

**DOI:** 10.1155/2011/848307

**Published:** 2011-11-14

**Authors:** B. L. Guilford, J. M. Ryals, D. E. Wright

**Affiliations:** Department of Anatomy and Cell Biology, University of Kansas Medical Center, Kansas City, KS 66160, USA

## Abstract

Emerging evidence suggests that dyslipidemia is an independent risk factor for diabetic neuropathy (DN) (reviewed by Vincent et al. 2009). To experimentally determine how dyslipidemia alters DN, we quantified neuropathic symptoms in diabetic mice fed a high-fat diet. Streptozotocin-induced diabetic C57BL/6 mice fed a high-fat diet developed dyslipidemia and a painful neuropathy (mechanical allodynia) instead of the insensate neuropathy (mechanical insensitivity) that normally develops in this strain. Nondiabetic mice fed a high-fat diet also developed dyslipidemia and mechanical allodynia. Thermal sensitivity was significantly reduced in diabetic compared to nondiabetic mice, but was not worsened by the high-fat diet. Moreover, diabetic mice fed a high-fat diet had significantly slower sensory and motor nerve conduction velocities compared to nondiabetic mice. Overall, dyslipidemia resulting from a high-fat diet may modify DN phenotypes and/or increase risk for developing DN. These results provide new insight as to how dyslipidemia may alter the development and phenotype of diabetic neuropathy.

## 1. Introduction 

Diabetic neuropathy (DN) is a principal chronic complication of both type 1 and type 2 diabetes and affects over half of diabetic patients [1–3]. Distal symmetric sensorimotor polyneuropathy, the most common and widely recognized form of DN, is a diffuse neuropathy characterized by both sensory and motor nerve deficits; however sensory dysfunction is the predominant feature of this neuropathy [4]. Affected patients can experience a large spectrum of sensory symptoms including chronic numbness, altered sensitivity to pain or touch, and impaired proprioception [2, 5, 6]. There is no definitive cure for this debilitating disease and symptomatic treatments have shown limited success [7]. 

 A dying back-type distal axon degeneration is the common underlying feature associated with DN [8]. It is thought that nerve dysfunction and degeneration leads to sensorimotor deficits, reduced nerve conduction velocities, and decreased epidermal innervation, all of which are characteristic signs of DN in human patients and animal models [7, 9]. Although it is clear that hyperglycemia plays a key role in the development and progression of DN [4, 9–12], a combination of multiple etiologies is likely responsible for axonal degeneration leading to the various types of neuropathy in diabetic patients [4, 10, 11]. Despite extensive study of proposed mechanisms, it remains unclear why some patients develop insensate versus painful symptoms and how underlying pathological mechanisms determine DN progression and phenotype.

The prevalence of societal overweight, obesity, and physical inactivity continues to increase thus the influence of lifestyle-related metabolic variables has become increasingly important in terms of DN risk and progression. Data from several large clinical trials suggest that dyslipidemia, typically defined as high serum levels of low-density lipoprotein cholesterol (LDL-C), high triglycerides, and/or low levels of high-density lipoprotein cholesterol (HDL-C) [13], is a major independent risk factor for the development of DN (reviewed in [1]). In addition, dyslipidemia is associated with the onset and progression of neuropathy in both type 1 and type 2 diabetes (reviewed in [14–16]). Body mass index has also been independently associated with the incidence of neuropathy in type 1 diabetic subjects [17]. Most individuals with neuropathy associated with prediabetes have painful small-fiber sensory neuropathy, are obese, and have dyslipidemia [12, 18–21]. Moreover, results from a cross-sectional study of type 2 diabetic subjects revealed that the prevalence of DN was twofold higher in type 2 diabetic subjects with the metabolic syndrome [22], a condition characterized by dyslipidemia, obesity, and hyperglycemia [23]. In support of the clinical evidence, nondiabetic mice fed a high-fat diet exhibit dyslipidemia [24], increased body weight, and painful neuropathy characterized by mechanical allodynia, thermal hypoalgesia, and nerve conduction deficits [24, 25]. 

Although diet was not assessed in the clinical studies, it is plausible to suggest that diet may indirectly affect DN progression and phenotype since dyslipidemia and obesity in adult humans can often be attributed to excess energy and fat intake. Taken together, this evidence suggests that diet may modulate the progression and phenotype of DN, and these effects may be mediated in part by diet-induced dyslipidemia and/or excess body weight. Although neuropathy and pre-diabetes have been documented in C57Bl/6 mice fed a high-fat diet [24, 25], the effects of a high-fat diet in conjunction with type 1 diabetes have not been studied. Here, we report the effects of a high-fat diet on neuropathy progression and phenotype in streptozotocin- (STZ-) induced type 1 diabetic mice. 

## 2. Methods

### 2.1. Animals and Diet

Seven-week-old male C57Bl/6 mice were purchased from Charles River (Wilmington, Mass), housed two mice per cage under pathogen free conditions, and placed on a 12:12 h light/dark cycle in the research support facility at the University of Kansas Medical Center. All animals had ad libitum access to food and water and were fed a standard diet (8604; Harlan Teklad, Madison Wisconsin; 14% kcals from fat, 32% protein, and 54% carbohydrate) or high-fat diet (07011; Harlan Teklad; 54% kcals from fat comprised of lard and corn oil, 21% protein, and 24% carbohydrate). Animals in the high-fat diet group were fed the standard diet for 1 week prior to streptozotocin (STZ) injection and began the high-fat diet 3 hours post STZ injection. All protocols and procedures were approved by the University of Kansas Medical Center Animal Use and Care Committee.

### 2.2. Diabetes Induction

A single intraperitoneal STZ injection (180 mg/kg body weight; Sigma-Aldrich, St. Louis, Mo) in 10 mM sodium citrate buffer (pH 4.5) was administered to 8-week-old C57Bl/6 mice to induce diabetes. Eight-week-old nondiabetic C57Bl/6 mice were injected with 400 *μ*L vehicle (sodium citrate) buffer. All mice were fasted for 3 hours before and after STZ injection. Body weight and blood glucose (glucose diagnostic reagents, Sigma, St. Louis, MO) were monitored 1 week after STZ injection and every week thereafter. Mice with blood glucose levels of >300 mg/dL (>16 mmol/L) were considered diabetic. STZ-injected mice with blood glucose levels below 300 mg/dL were excluded from the study. Treatment groups are abbreviated throughout as follows: nondiabetic standard diet (NdStd); nondiabetic high-fat diet (NdHF); diabetic standard diet (DbStd); diabetic high-fat diet (DbHF).

### 2.3. Behavioral Testing

Behavioral testing to assess characteristic signs of DN including mechanical sensitivity, thermal sensitivity, and sensorimotor ability (beam walk task) were performed 1 week before STZ injection and subsequently thereafter. Before each behavioral test was performed for the first time, mice were acclimated to the apparatus during two separate training sessions. Immediately prior to each behavioral test, mice were acclimated to the behavior room for 30 minutes followed by a 30-minute acclimation on the testing apparatus (except beam walk).

#### 2.3.1. Mechanical Sensitivity

Mice were placed on an elevated wire mesh screen (55 cm above table), enclosed individually in clear plastic cages (11 × 5 × 3.5 cm), and mechanical sensitivity was assessed using a 1.4 g von Frey monofilament (Stoelting, Wood Dale, IL) which was applied 6 times to each hind paw footpad. A combined mean percent withdrawal from a total of 12 applications was calculated per mouse and used to calculate group means.

#### 2.3.2. Thermal Sensitivity

Mice were placed on a Hargreaves apparatus, and 4.0 V radiant heat source was applied four times to each hind paw footpad. Time elapsed before the animal withdrew the paw was recorded as withdrawal latency. A combined mean withdrawal latency (secs) was calculated from a total of 8 applications per mouse and used to calculate group means.

#### 2.3.3. Beam Walk

Mice were trained to traverse a 1 m-long, 1.2 cm diameter, wooden beam (adapted from [26, 27]). As each animal crossed the beam, the number of times the right or left paw slipped off the beam was counted as a footslip. This behavioral test was recorded using a digital video camera. The combined mean number of footslips/mouse was calculated from a total of 3 trials per session and used to calculate group means.

### 2.4. Nerve Conduction Velocity

At 8 weeks post-STZ and immediately before sacrifice, mice were deeply anesthetized by intraperitoneal injection with Avertin (1.25% v/v tribromoethanol [Sigma-Aldrich], 2.5% tert-amyl alcohol [Sigma-Aldrich], dH_2_O; 250 mg/kg) and motor and sensory nerve conduction velocities were recorded according to Stevens et al. [28] and as described previously by Muller et al. [29]. Body temperature was monitored by rectal probe and maintained at 37°C. 

### 2.5. Serum Lipids and Insulin

At 8 weeks after STZ injection and immediately following nerve conduction velocity (NCV) studies, anesthetized mice were sacrificed. Blood was collected into Eppendorf tubes, placed on ice to clot for 30 minutes, and centrifuged for 15 minutes at 3,000 xg. Serum was removed and frozen at −80°C. Serum samples were assayed for total cholesterol (Cholesterol Total E kit, Wako Diagnostics) LDL-C (L-type LDL-C kit, Wako Diagnostics), triglycerides (triglyceride kit, Cayman Chemical), and insulin (mouse insulin Elisa, Alpco).

### 2.6. Immunohistochemistry

Anesthetized mice were sacrificed, and tissues were dissected at 8 weeks after STZ injection. Unfixed lumbar dorsal root ganglia (DRG) were dissected and frozen in Tissue-Tek O.C.T. Compound (OCT, Sakura, Torrance, Calif). Hind paw footpads were dissected and fixed for 2 hours in Zamboni's fixative (4% formaldehyde, 14% saturated picric acid, 0.1 M phosphate-buffered saline [PBS, pH 7.4] at 4°C), immersed overnight in 1% PBS (pH 7.4 at 4°C), and finally immersed for 4 hours in 30% sucrose in 1X PBS (pH 7.4 at 4°C). After freezing in OCT, DRG and footpads were sectioned on a cryostat at 10 *μ*m and 30 *μ*m, respectively, mounted on Superfrost Plus slides (Fisher Scientific, Pittsburgh, Pa), and stored at 4°C.

After thawing for 5 minutes at room temperature, slide-mounted tissue was covered with blocking solution (0.5% porcine gelatin, 1.5% normal donkey serum, and 0.5% Triton-X in Superblock buffer; Pierce) for 1 hour at room temperature. Slides were then incubated overnight at 4°C in primary antibody diluted in blocking solution. Slides were washed the following day for 2 × 10 min with PBST followed by 3-hour incubation with fluorochrome-conjugated secondary antibodies diluted in PBS and blocking solution. Following 2 × 10 min washes in 1X PBS, slides were rinsed in deionized distilled H_2_O, coverslipped and stored at −20°C (footpad sections) or cover slipped with Anti-Fade Prolong Gold (Invitrogen, Carlsbad, Calif), and stored at room temperature (DRG sections).

### 2.7. Intraepidermal Nerve Fiber Density

Rabbit anti-PGP 9.5 (1 : 400; Chemicon, Temecula, Calif) and Alexa-488 (1 : 2000; Molecular Probes, Eugene, Ore) were used to label and visualize dermal and epidermal nerve fibers. Fluorescent digital images were acquired from epidermal regions using a Nikon Eclipse E800 microscope. The number of nerve fibers per section that cross the epidermal-dermal border was counted using a 40x objective in order to visualize fibers throughout the full depth of the tissue section. NIH Image J software was used to measure each epidermal region and intraepidermal nerve fiber density (IENFD) was expressed as number of fibers per millimeter of epidermis. The combined mean IENFD from 6 images per mouse was used to calculate group means. 

### 2.8. Oxidative Stress

Primary antibodies (rabbit anti-nitrotyrosine [1 : 1000; Chemicon, Temecula, Calif] and rabbit anti-neurofilament H [1 : 10,000, Chemicon, Temecula, Calif]) and fluorescent secondary antibodies (Alexa 488 and Alexa 555 [both 1 : 2000; Molecular Probes, Eugene, Ore]) were used to label and visualize nitrated tyrosine residues and neurons, respectively, in the lumbar DRG. Six fluorescent digital images from 6 DRG sections per mouse were acquired at 40x using a Nikon Eclipse E80i microscope. All pictures were taken using the identical exposure time. Metamorph software (MDS Analytical Technologies, Downingtown, Pa) was used to circle individual neurons and quantify the average intensity of nitrotyrosine fluorescence and total area per neuron. To ensure that each cell circled included the area for the entire neuron, only neurons with a visible central nucleus were included in the analysis. For each image, background intensity was measured from a region adjacent to the tissue. The background intensity for each image was subtracted from the average fluorescence intensity for each neuron from that image. Group means for average nitrotyrosine fluorescence per cell were calculated for 4 different neuron sizes based on the following areas: 1–200 *μ*m^2^, 201–400 *μ*m^2^, 401–600 *μ*m^2^, and ≥601 *μ*m^2^. 

### 2.9. Statistics

Data were analyzed using a two-factor analysis of variance (ANOVA) or repeated measures ANOVA with Fisher's test of least square difference post hoc comparisons. Statistical significance was set at *P* < 0.05.

## 3. Results

### 3.1. Body Weight, Glucose, and Insulin

**Figure 1:**
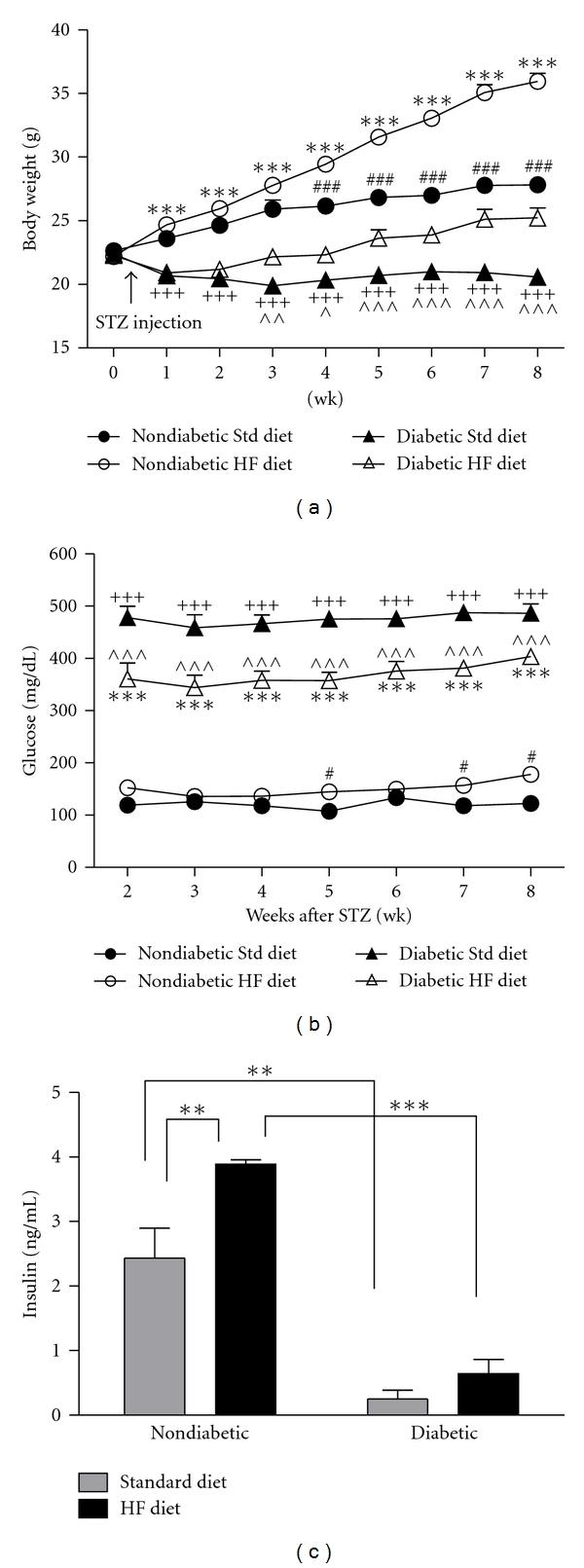
Body weight (a) and blood glucose (b) (*n* = 14–21 mice per group). ^#^ *P* < 0.05 and ^ ###^ *P* < 0.001: NdStd versus NdHF; ***P < 0.001: NdHF versus DbHF; ^+++^ *P* < 0.001: NdStd versus DbStd; ^∧^ *P* < 0.05, ^∧∧^ *P* < 0.01, and ^∧∧∧^ *P* < 0.001: DbStd versus DbHF. (c) Serum insulin (*n* = 3–8 mice per group). *P < 0.05; **P < 0.01; ***P < 0.001. Data are presented as means ±SEM.

Diabetic mice displayed common symptoms of diabetes, including polydipsia and polyuria within 1 week after STZ injection. Typical with this model, diabetic mice weighed less than their nondiabetic counterparts. However, high-fat-fed diabetic mice gained weight (+3 g) compared to diabetics fed the standard diet who lost 1.7 g on average (Figure 1(a)). Nondiabetic mice on the standard diet gradually gained 5 g in body weight, whereas high-fat-fed nondiabetic mice gained 13 g over the course of the 8-week study (Figure 1(a)). Energy intake was not different among nondiabetic groups (NdStd 13.5 ± 1.3 kcals/day; NdHF 14.0 ± 0.5 kcals/day, *P* > 0.05 for NdStd versus NdHF). In contrast, diabetic mice on the standard diet consumed at least 2 times as many kcals/day compared to all other groups (DbStd 29.7 ± 1.8 kcals/day; DbHF 15.2 ± 7.8 kcals/day, *P* < 0.05 for DbStd versus NdStd, NdHF, and DbHF). 

 In nondiabetic mice, high-fat feeding resulted in a mild increase in blood glucose levels compared to nondiabetic mice on the standard diet that maintained their blood glucose levels around 120 mg/dL throughout the 8-week study (Figure 1(b)). As early as 1 week following STZ injection, diabetic mice had significantly higher blood glucose levels compared to their nondiabetic counterparts (Figure 1(b)). However, the combination of diabetes and the high-fat diet resulted in significantly lower blood glucose levels compared to diabetic mice on the standard diet, but glucose levels were still higher than both nondiabetic groups (Figure 1(b)). Insulin levels were significantly lower in both diabetic groups compared to the nondiabetic groups on the equivalent diet (Figure 1(c)). Finally, high-fat feeding induced hyperinsulinemia in nondiabetic mice (Figure 1(c)).

### 3.2. A High-Fat Diet Induces Dyslipidemia

**Figure 2:**
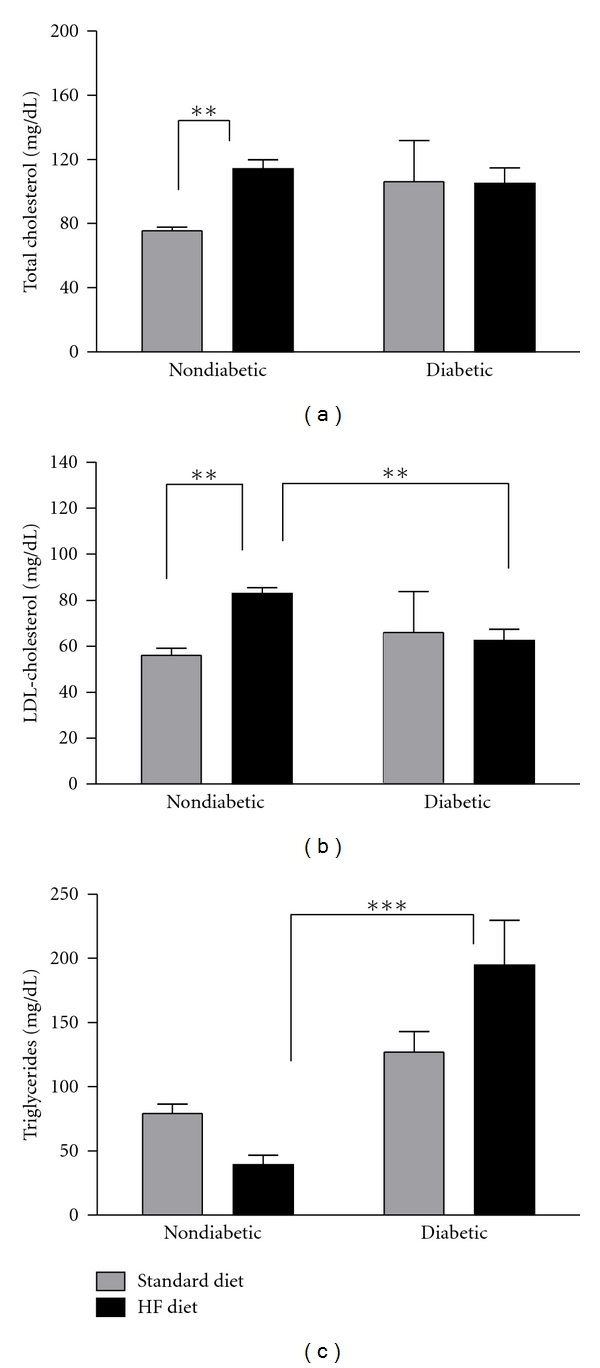
Serum Lipids. Blood was collected at 8 weeks following STZ from non-fasted mice. Total Cholesterol (a), LDL-Cholesterol (b), and Triglycerides (c). Data are presented as means ± SEM (*n* = 3–8 mice per group). **P < 0.01 and ***P < 0.001.

Nondiabetic mice fed a high-fat diet had higher total cholesterol levels compared to nondiabetic mice fed a standard diet (Figure 2(a)). However, diabetes did not significantly alter total cholesterol in mice fed the standard or high-fat diet compared to their nondiabetic counterparts (Figure 2(a)). The high-fat diet increased LDL-C in nondiabetic mice, which mirrored the effect of high-fat feeding on total cholesterol in nondiabetic mice (Figures 2(a) and 2(b)). Diabetes did not alter LDL-C levels in mice fed the standard diet compared to their nondiabetic counterparts (Figure 2(b)). Triglyceride levels were not significantly affected by the high-fat diet in nondiabetic mice (Figure 2(c)). Diabetic mice on a standard diet had slightly higher triglycerides compared to their nondiabetic counterparts, while triglycerides were significantly higher in the diabetic high-fat-fed group compared to the nondiabetic high-fat-fed group (Figure 2(c)).

### 3.3. Sensorimotor Behavior Is Altered by High-Fat Feeding

**Figure 3:**
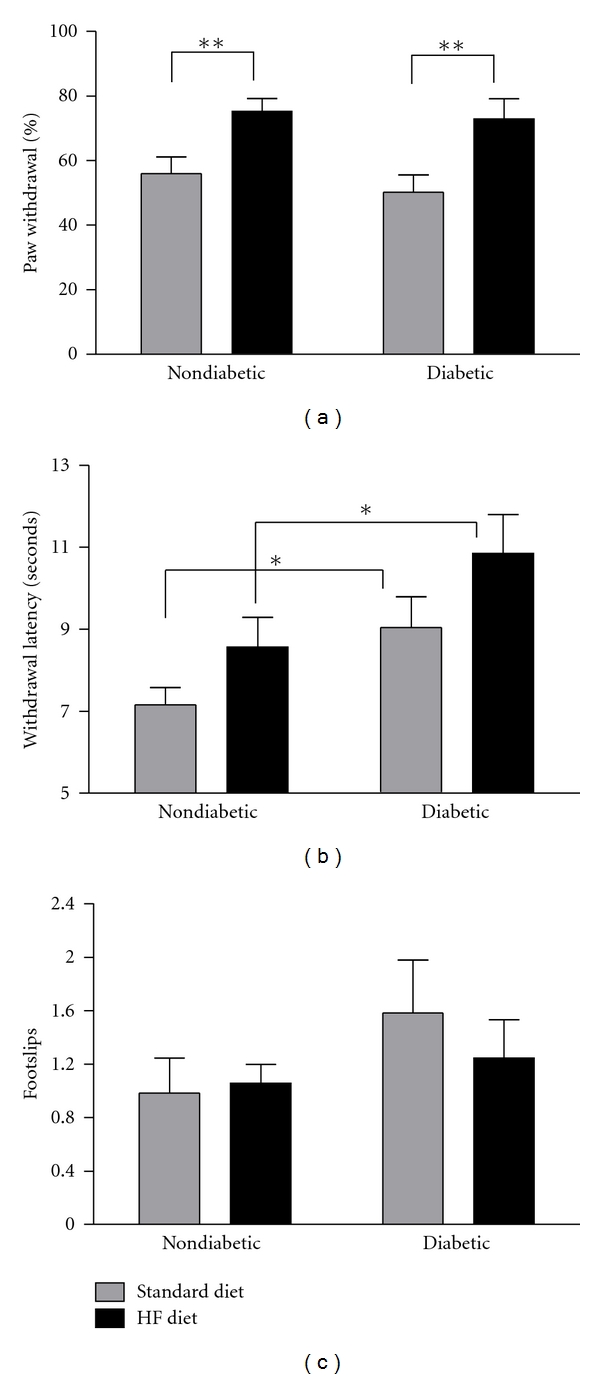
Sensorimotor Behavior. (a) Mechanical sensitivity was assessed using 12 repeated applications of a 1.4 g von Frey monofilament (*n* = 15–21 mice per group). (b) Thermal sensitivity. Mice were placed on a Hargreaves apparatus, and a 4.0 V radiant heat source was applied to the hind paw footpad. The time elapsed before the animal withdrew the paw was recorded as withdrawal latency (*n* = 6–13 mice per group). (c) Sensorimotor ability was assessed by quantifying mean hind paw footslips as mice traversed a narrow wooden beam (*n* = 6–13 mice/group). Data are from 8 weeks after STZ and presented as means ±SEM. *P < 0.05 and **P < 0.01.

Previous studies of this murine strain have demonstrated that STZ-induced diabetic C57Bl/6 mice can develop a slowly progressive insensate neuropathy characterized by a reduction in mechanical sensitivity that appears after 4 weeks of diabetes. However, the degree to which individual cohorts of mice develop a loss of sensation has been variable, ranging from 27–50% change in sensitivity, and we have never observed mechanical allodynia with this model, mouse strain, and diet. [30–35]. In the current cohort of mice, mechanical sensitivity was reduced by 11% (Figure 3(a)); however, the pattern of mechanical sensitivity remained similar to previous studies that document STZ diabetes-induced mechanical hypoalgesia. In striking contrast, the high-fat diet increased mechanical sensitivity in both nondiabetic and diabetic mice compared to their counterparts fed a standard diet by 35% and 45%, respectively (Figure 3(a)). Diabetes significantly reduced thermal sensitivity in mice on the standard diet but was not significantly worsened by the addition of the high-fat diet (Figure 3(b)). However, diabetic mice fed the high-fat diet had significantly lower thermal sensitivity than high-fat-fed nondiabetic mice (Figure 3(b)). A beam walk task was used to assess sensorimotor ability at 8 weeks following STZ. The number of hind paw slips measured while mice traversed a wooden beam was used to assess sensorimotor deficits, and the number of slips was not significantly different among any groups (Figure 3(c)).

### 3.4. Nerve Conduction and Epidermal Innervation

**Figure 4:**
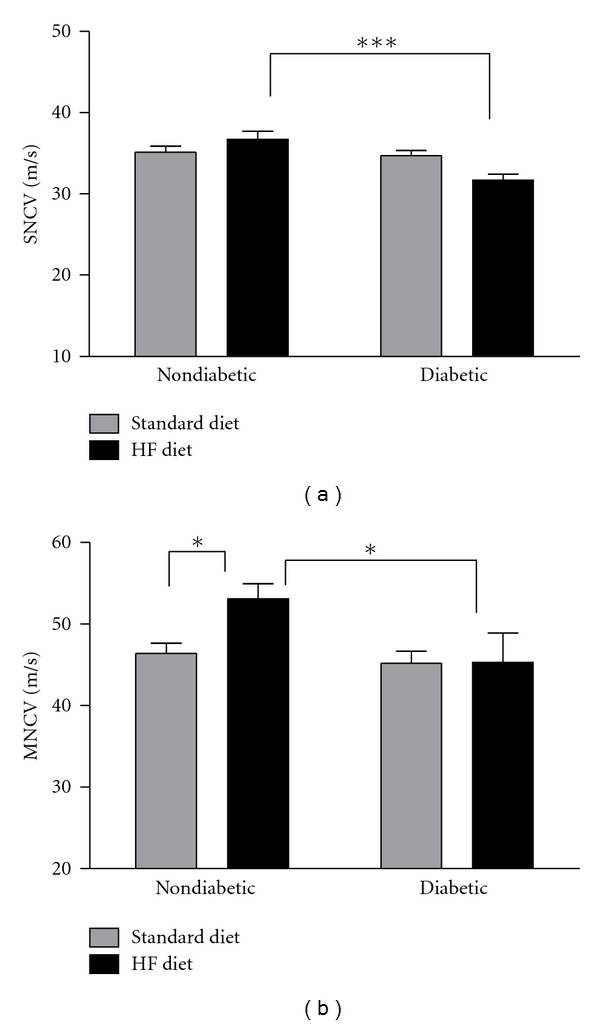
Sensory nerve conduction velocity (a) and motor nerve conduction velocity (b) were measured at 8 weeks after STZ. Data are presented as means ± SEM (*n* = 3–8 mice per group). *P < 0.05 and ***P < 0.001.

**Figure 5:**
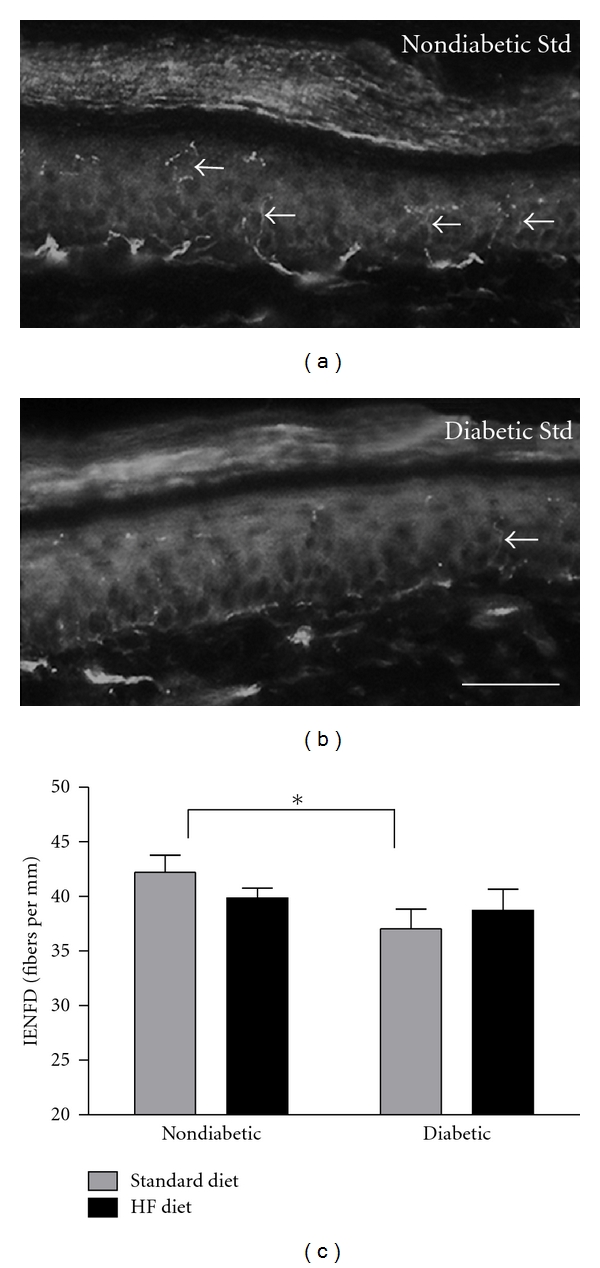
Intraepidermal Nerve Fiber Density. Plantar skin of the hindpaw was dissected at 8 weeks following STZ. Immunofluorescence staining for PGP 9.5 was used to visualize and count nerve fibers that cross the epidermal/dermal border. (a-b): Representative images showing a section of plantar skin used to quantify IENFD. (a) Nondiabetic Standard Diet. (b) Diabetic Standard Diet. Arrows indicate individual axons within the epidermis. Scale bar = 50 *μ*M. (c) Quantification of IENFD (*n* = 11-12 mice per group). Data presented as means ± SEM. *P < 0.05.

Neither diabetes nor high-fat feeding alone altered sensory nerve conduction velocity (Figure 4(a)). Conversely, the combination of diabetes and high-fat feeding significantly reduced sensory and motor nerve conduction velocities compared to nondiabetic mice fed the high-fat diet (Figures 4(a) and 4(b)). High-fat feeding increased motor nerve conduction velocity (MNCV) in nondiabetic mice, but MNCV was not significantly affected by diabetes alone (Figure 4(b)). Consistent with previous studies documenting decreased cutaneous innervation associated with DN [30–32, 36–40], IENFD was significantly reduced in the hind paw footpad skin after 8 weeks of diabetes (Figures 5(a)–5(c)). However, the combination of the high-fat diet and diabetes did not significantly alter IENFD (Figure 5(c)).

### 3.5. Oxidative Stress

**Figure 6:**
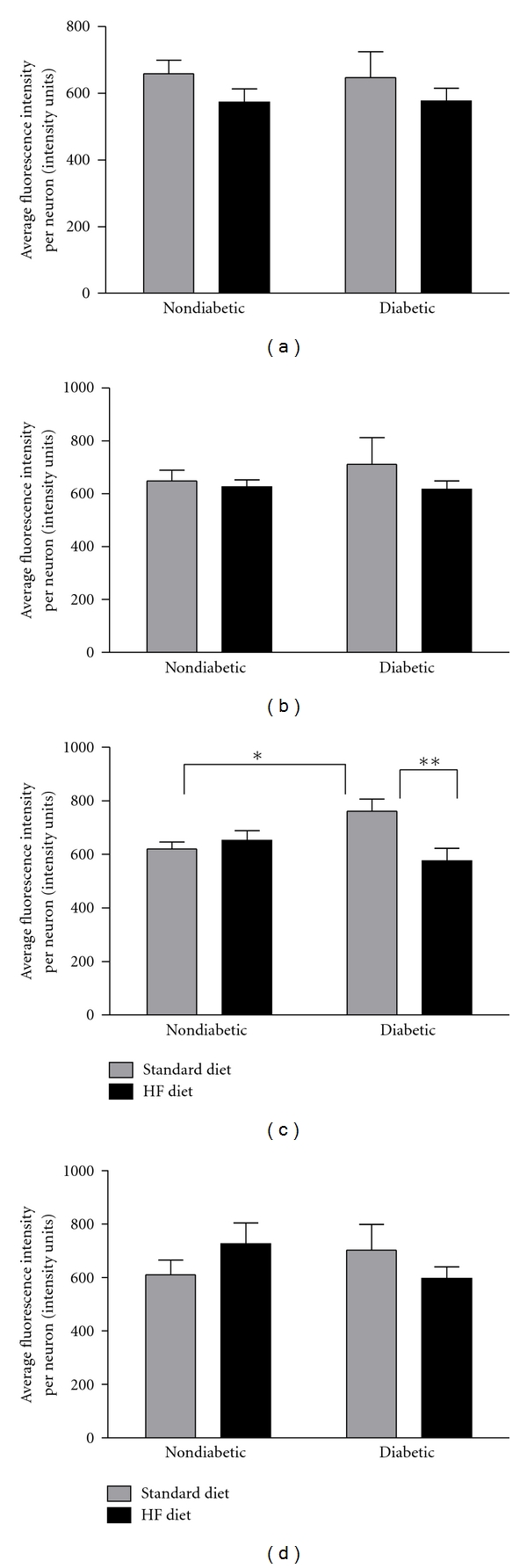
Nitrotyrosine expression in lumbar DRG neurons. Quantification of average nitrotyrosine fluorescence intensity per neuron in the lumber DRG. Mean fluorescence intensity was calculated for a total of approximately 10 cells per image from six images per mouse and group means were calculated with respect to cell size. Group means for average nitrotyrosine fluorescence per cell are binned by neuron size according to area^2^: (a) 0–200 *μ*m^2^, (b) 201–400 *μ*m^2^, (c) 401–600 *μ*m^2^, and (d) ≥601 *μ*m^2^. Data are presented as means ± SEM (*n* = 3–8 mice per group). *P < 0.05 and **P < 0.01.

**Figure 7:**
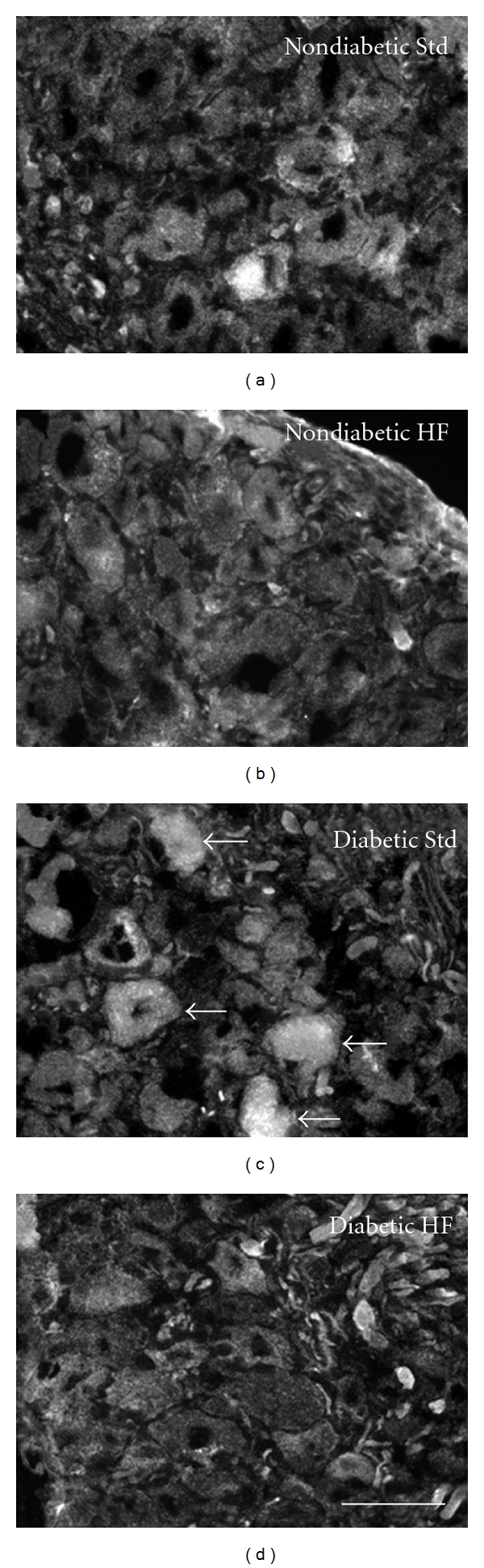
Representative images showing nitrotyrosine expression in the lumbar DRG from mice in each of four groups: (a) nondiabetic standard diet, (b) nondiabetic high-fat diet, (c) diabetic standard diet, and (d) diabetic high-fat diet. Arrows indicate medium-sized neurons expressing high levels of nitrotyrosine. Scale bar = 50 *μ*M.

Immunohistochemistry was used to quantify the abundance of nitrotyrosine, a marker of oxidative stress, in the lumbar DRG with respect to neuron size. Neither diabetes nor the high-fat diet significantly altered levels of nitrated tyrosine residues in small (Figures 6(a) and 6(b)) or large DRG neurons (Figure 6(d)). However, nitrotyrosine fluorescence was significantly increased in medium-sized neurons in diabetic mice fed the standard diet compared to their nondiabetic counterparts (Figures 6(c) and 7). In addition, nitrotyrosine fluorescence was significantly increased in diabetic mice fed the standard diet compared to diabetic mice fed the high-fat diet (Figure 6(c)).

## 4. Discussion

Recent evidence indicating that dyslipidemia may increase a diabetic patient's risk of developing neuropathy (reviewed in [1]), taken in conjunction with high-fat feeding studies in rodents [24, 25, 41], suggests diet may play an important role in modulating DN progression and phenotype. Here, our data reveals that a high-fat diet fed to STZ-induced diabetic C57BL/6 mice significantly alters the phenotype of neural symptoms by inducing a painful neuropathy (mechanical allodynia) instead of an insensate neuropathy (mechanical insensitivity) that often develops in these diabetic mice [30–33]. In addition, our results support clinical data (reviewed in [1]), that suggests that dyslipidemia may be an independent risk factor for development of DN.

In numerous studies using this murine strain, STZ dose, and standard diet, our laboratory and others have reported that STZ-induced diabetic C57Bl/6 mice develop a slowly progressive insensate neuropathy characterized by a reduction in mechanical sensitivity after 4 weeks of diabetes [30–33, 40]. It is important to note that our studies with this inbred strain differ from others that have reported mechanical allodynia [42–45]. One important issue to help explain these differences is that laboratories reporting mechanical hypoalgesia obtained C57Bl/6 mice from Charles River or Harlan Laboratories [30–35] while laboratories reporting mechanical allodynia obtained C57Bl/6J mice from Jackson Laboratories [42–45]. In addition, mice from studies reporting mechanical allodynia were fed an unspecified standard mouse chow (PMI Nutrition International) during the study while mice from studies reporting mechanical hypoalgesia were fed a standard diet (Harlan Teklad 8604 or Purina 5001). Although both are standard diets, antioxidant and macronutrient composition varies significantly between vendors; thus it is possible that dietary differences may in part contribute to the dichotomous neuropathy phenotype observed in this mouse strain. It is plausible to suggest that diabetic neuropathy may manifest differently due to varied composition of the diet on which the animals were raised and maintained. The current findings are consistent with this discrepancy and point to an important role of the diet related to mechanical sensitivity and genetic background. 

 In the current study, we observed a slight reduction in mechanical sensitivity, although the reduction in mechanical sensitivity did not reach statistical significance after 8 weeks of diabetes in this cohort of animals. Importantly, however, STZ-diabetic mice fed a high-fat diet displayed mechanical allodynia, as opposed to the insensate phenotype previously observed in this inbred mouse strain. The high-fat diet induced a robust mechanical allodynia as mechanical sensitivity was increased by 35% and 45%, respectively, in nondiabetic and diabetic mice compared to their counterparts fed a standard diet. This finding is particularly important because it suggests that diet modulates DN phenotype in rodents thus dietary manipulation could be used as a new tool to investigate mechanisms that cause some patients to experience insensate symptoms while others have painful symptoms. In addition, this data is consistent with previous studies that reported that a high-fat diet induces neuropathy in nondiabetic mice [24, 25].

Diet-induced changes in neuropathy were not uniform across modalities or symptoms. For example, diabetes significantly reduced epidermal innervation as previously noted [30–32]; however, the high-fat diet alone or in combination with diabetes did not further affect IENFD. Similarly, impaired thermal sensitivity was not exacerbated by the high-fat diet in STZ-diabetic mice. This suggests that diabetes alone may be the driving factor underlying reduced IENFD and thermal hypoalgesia in this model.

Neither diabetes nor the high-fat diet significantly altered gait and balance as assessed by the beam walk task. Body weight differences due to the high-fat diet appeared to affect the beam walk task that potentially confounded this data, increased error, and reduced our ability to detect statistically significant differences in hind paw footslips. In addition, gait and balance deficits detected by a beam walk task are thought to be, in part, attributed to altered muscle spindle group Ia innervation [29], which may not be affected after only 8 weeks of diabetes.

Although sensory and motor NCV were reduced in diabetic mice fed the high-fat diet compared to their nondiabetic counterparts, diabetes alone did not significantly affect NCV in the current study. Sensory and motor nerve conduction deficits are a well-documented consequence of diabetes in rodents [28, 43, 44, 46–53] and several studies report diabetes-induced slowed SNCV and MNCV in STZ-diabetic C57Bl/6 mice. However, reports of diabetes-induced nerve conduction deficits can be variable, ranging from no change in SNCV [29, 34, 35] or MNCV [54, 55] up to approximately 30% and 20% reductions in sensory and motor NCV, respectively [52]. Moreover, the time points examined are important too, as onset of deficits has been reported to begin as early as 3 weeks after STZ [49] or as late as 3 months after STZ [56]. 

It is unclear why the results from the current study specifically differ from many other previous reports of slowed NCV in STZ-diabetic C57Bl/6 mice of similar age and diabetes duration [43, 44, 49, 50], but a major contributing factor may be the small number of animals examined, or differences in antioxidant and macronutrient composition of the standard diet, or even the mouse supplier (Charles River versus Jackson Laboratories). As mentioned above, dietary conditions may strongly impact the neuropathy phenotype. For example, previous reports of slowed NCV in STZ-diabetic C57Bl/6 mice of similar age and diabetes duration obtained mice from Jackson Laboratories or other vendors [43, 44, 49, 50] while the C57Bl/6 mice in the current study were obtained from Charles River. In addition, the diet the animals were fed during the previous studies [43, 44, 49] was an unspecified standard mouse chow from PMI Nutrition International while the standard diet in the current study was from Harlan Teklad (8604) and were likely different in antioxidant and macronutrient composition. 

Consistent with findings from the current study, other previous studies did not detect decreases in SNCV in STZ-diabetic C57Bl/6 mice, but in contrast to the current results, showed diabetes-induced slowed MNCV [29, 34, 35]. However, diabetes duration was longer in the previous studies (10, 12, or 16 weeks) than the current study (8 weeks). Consistent with our current findings of NCV at 8 weeks after STZ, Ramji et al. reported no significant differences in SNCV and MNCV at 2 months following STZ, and significant SNCV and MNCV deficits were not observed until 3 months after STZ in diabetic mice [56]. 

Nondiabetic mice fed the high-fat diet had significantly increased MNCV compared to nondiabetic mice fed the standard diet. In contrast, previous studies have reported significant SNCV and MNCV deficits after high-fat feeding in C57Bl/6 mice [24, 25, 41]. However, the duration of high-fat feeding was longer (12 or 16 weeks) in the previous studies [24, 25, 41] compared to 8 weeks of high-fat feeding in the current study. Although MNCV was significantly increased in nondiabetic mice fed the high-fat diet compared to their counterparts on the standard diet, MNCV in high-fat-fed nondiabetic mice was 53.1 ± 1.9 in the current study which is similar to previous reports of MNCV (approximately 50 m/s) in nondiabetic mice fed a standard diet [29, 43, 44, 52]. Although no known reports of high-fat feeding have shown increased MNCV in nondiabetic mice, it is possible that increased fat intake during a critical stage of development (8–16 weeks) may affect myelination and transiently increase MNCV at this specific stage of development (16 weeks of age).

 Behavioral responses to sensorimotor tests incorporate inputs from multiple axonal fiber types within peripheral nerves. The von Frey method of assessing mechanical sensitivity stimulates a combination of large myelinated A-beta fibers that are sensitive to light touch and small unmyelinated C fibers that are pain responsive. The Hargreaves test stimulates pain and temperature-sensing C and A-delta fibers, whereas the beam walk test likely assesses contributions from dermal A-fibers and muscle afferent fibers innervating muscle spindles, both of which are important for gait, balance, and proprioception. IENFD is an excellent tool for quantifying small fiber loss, is often used when diagnosing DN humans, and has been shown to correlate with diabetes duration in human patients [9, 29, 36, 57]. Slowed SNCV has been thought to reflect conduction deficits predominately in large fibers [29], but reports documenting diabetes-induced slowed SNCV vary as some studies report decreases [58] while others report no change [29, 54]. The diabetic environment affects each fiber population differently and the type of fibers predominantly affected varies with mouse strain and in humans.

In the current study, the high-fat diet induced mechanical allodynia, but diabetes-induced deficits in thermal sensitivity were not significantly worsened by the addition of the high-fat diet. Neither diabetes nor the high-fat diet significantly altered gait and balance as assessed by the beam walk task. Apart from the robust effects of the high-fat diet on mechanical sensitivity, none of the other behavioral data nor anatomical or physiological assessments performed here suggest that the high-fat diet affects sensorimotor behavior or nerve morphology and function differently than diabetes alone. It will be important to investigate how a high-fat diet alters mechanical sensitivity, and whether this selective effect is related to specific peripheral fiber-type or central processing of mechanical sensitivity in the CNS.

Here, we report that a high-fat diet significantly increased body weight in nondiabetic and diabetic mice compared to their counterparts on a standard diet, suggesting that the metabolic derangements that accompany diet-induced obesity may have harmful effects on nerve fiber function, thereby leading to mechanical allodynia.

In addition, total cholesterol and LDL-C were significantly increased in high-fat-fed nondiabetic mice compared to their nondiabetic counterparts on the standard diet, and triglyceride levels were significantly higher in STZ-diabetic mice fed the high-fat diet compared to their nondiabetic counterparts on the high-fat diet. Because at least one parameter of the lipid profile (serum total cholesterol, LDL-C, or triglycerides) was increased in high-fat-fed nondiabetic and STZ-diabetic mice, both groups could be considered dyslipidemic. Thus, diet-induced dyslipidemia may be an underlying factor that drives mechanical allodynia in rodents. Consistent with clinical evidence suggesting that dyslipidemia is associated with DN onset and progression (reviewed in [14–16]), our data supports the view that diet-induced dyslipidemia may induce neuropathy in prediabetic patients and modulate DN onset, progression, and/or phenotype.

In the current study, nondiabetic mice fed the high-fat diet exhibited pre-diabetes characterized by elevated glucose levels and hyperinsulinemia. As expected, glucose was significantly higher in both diabetic groups compared to their nondiabetic counterparts. However, glucose levels were lower in high-fat-fed diabetics compared to diabetic mice on the standard diet which is most likely explained by the lower percentage of total kilocalories derived from carbohydrate in the high-fat diet (24%) versus the standard diet (40%). STZ is a selective beta islet cell toxin that kills the insulin-producing cells in the pancreas thus, as expected, insulin levels were significantly lower in both diabetic groups compared to their nondiabetic counterparts. Although high-fat feeding induced mechanical allodynia in both nondiabetic and diabetic mice, nondiabetic mice were hyperinsulinemic whereas diabetic mice remained hypoinsulinemic after 8 weeks of high-fat feeding. Furthermore, diabetic mice on the high-fat diet had significantly higher glucose levels than their nondiabetic counterparts, but mechanical sensitivity was not different between these groups. Thus, our data suggests that glucose and insulin levels may not be key factors driving high-fat diet-induced mechanical allodynia observed in the current study. 

A high-fat diet and diabetes alone have consistently been shown to increase oxidative stress in rodents [24, 41, 59–61], and increased oxidative stress has been proposed as a mechanism that contributes to pathogenesis of DN (reviewed in [10, 62, 63]). Vincent and colleagues reported that dyslipidemia leads to high levels of oxidized low-density lipoproteins (ox-LDLs) in mice and *in vitro*, and oxLDLs lead to severe DRG neuron oxidative stress and neuron injury, thus identifying a potential mechanism by which dyslipidemia may contribute to the development of DN [24]. 

In addition, the nitrosative component of free radical and oxidant-induced injury in STZ diabetes has been well characterized with several previous studies reporting increased nitrotyrosine levels in DRG or spinal cord neurons and/or sciatic nerve in STZ-diabetic mice [43–45, 50] and rats [51, 64, 65]. In the current study, nitrotyrosine levels in medium-sized DRG neurons (401–600 *μ*m^2^) were significantly increased in diabetic mice fed the standard diet compared to their nondiabetic counterparts. In addition, nitrotyrosine fluorescence was significantly increased in diabetic mice fed the standard diet compared to diabetic mice fed the high-fat diet, suggesting that the high-fat diet did not exacerbate elevated nitrotyrosine levels beyond effects from diabetes in this neuronal population. Nitrotyrosine levels in small or large neurons were not significantly altered by diabetes. Although previous studies have not measured nitrotyrosine with respect to neuron size, our results support the current literature suggesting that nitrosative stress is increased in DRG neurons of STZ-diabetic mice [42–45]. 

Although diabetes alone significantly increased nitrotyrosine levels in medium-sized DRG neurons, high-fat feeding alone did not significantly alter nitrotyrosine levels in nondiabetic or diabetic mice. In contrast, previous studies report that nitrotyrosine is significantly increased in sciatic nerve after 16 weeks of high-fat feeding in C57Bl/6 mice [25, 41]. However, it should be noted that these previous studies assessed nitrotyrosine in the sciatic nerve after 16 weeks of high-fat feeding [25, 41] while the current study measured nitrotyrosine in DRG neurons after only 8 weeks of high-fat feeding. It is plausible to suggest that during short-term high-fat feeding (i.e., 8 weeks), compensatory mechanisms to mediate high-fat diet-induced oxidative stress are upregulated but these mechanisms become overwhelmed as high-fat feeding becomes longer term. These results should be interpreted with caution because we only quantified one oxidative stress marker in one tissue (DRG), and this was a short-term study that lasted only 8 weeks. Although nitrotyrosine levels were not affected by the high-fat diet alone in the DRG, it is possible that the effects of diabetes and/or the high-fat diet may have been more apparent if we had quantified additional oxidative stress markers (i.e., hydroxyoctadecadienoic acid, 4-hydroxynonenal, dityrosine) in the DRG or other tissues such as plasma, isolated LDLs, or sciatic nerve. 

Little is known about the mechanisms contributing to high-fat diet-induced mechanical allodynia, but one recent study indicates that an extract from the Artemisia plant (known for its anti-inflammatory and antinociceptive properties) alleviates high-fat diet-induced mechanical allodynia and reduces 12/15 lipoxygenase (regulates proinflammatory cytokine production) upregulation, suggesting that inflammation may play a role in high-fat diet-induced neuropathy [41]. Proinflammatory cytokines and chemokines have been widely implicated in chronic pain and are thought to contribute to the central sensitization that results in mechanical allodynia [66–69]. Importantly, obesity is associated with chronic low-grade inflammation [70, 71], and a high-fat diet increases the proinflammatory cytokines IL1-*β*, tumor necrosis factor alpha (TNF-*α*), C-reactive protein (CRP), and IL1-6 and stimulates inflammatory signaling in adipose, serum, liver, and brain [72–76]. Along with glia and macrophages [77–82], adipose is an important source of proinflammatory cytokines that may upregulate cytokine production due to increased adipose resulting from a high-fat diet [72, 73]. Therefore, a proinflammatory environment in the spinal cord may be a key mechanism in the development of mechanical allodynia associated with diabetes and/or a high-fat diet. Future studies will investigate the role of spinal inflammation in high-fat diet-induced mechanical allodynia. 

## 5. Conclusions

These results demonstrate that a high-fat diet fed to STZ-induced diabetic C57BL/6 mice significantly alters the phenotype of neural symptoms by inducing a painful neuropathy (mechanical allodynia) instead of the insensate neuropathy (mechanical insensitivity) that we have previously observed in this inbred strain. In addition, our results support previous studies [24, 25] that show that a high-fat diet fed to nondiabetic mice can induce pre-diabetes and neuropathy. In conclusion, dyslipidemia resulting from a high-fat diet may modify DN phenotype and/or increase risk for developing DN. Furthermore, the metabolic derangements that accompany diet-induced obesity may have harmful effects on nerve fiber function thereby leading to mechanical allodynia. These results may provide some insight into why some patients develop painful versus insensate neuropathy. 

 Although the ability of a high-fat diet to induce mechanical allodynia is novel, little is known about the mechanisms responsible for this robust painful DN phenotype in this model thus further study is warranted. Positive outcomes from future studies could lead to dietary modifications and/or increased use of treatments that improve dyslipidemia (i.e., omega 3 fatty acid supplementation, statins, or exercise) as therapeutic interventions for patients with painful diabetic neuropathy.
